# Kinase shRNA screening reveals that TAOK3 enhances microtubule-targeted drug resistance of breast cancer cells via the NF-κB signaling pathway

**DOI:** 10.1186/s12964-020-00600-2

**Published:** 2020-10-21

**Authors:** Tsung-Ching Lai, Chih-Yeu Fang, Yi-Hua Jan, Hsiao-Ling Hsieh, Yi-Fang Yang, Chun-Yu Liu, Peter Mu-Hsin Chang, Michael Hsiao

**Affiliations:** 1 Division of Pulmonary Medicine, Department of Internal Medicine, Wan Fang Hospital, Taipei Medical University, Taipei, 116 Taiwan; 2 Pulmonary Research Center, Wan Fang Hospital, Taipei Medical University, Taipei, 116 Taiwan; 3 Genomics Research Center, Academia Sinica, Taipei, 115 Taiwan; 4 Department of Medical Education and Research, Kaohsiung Veterans General Hospital, Kaohsiung, 81362 Taiwan; 5 Department of Oncology, Taipei Veterans General Hospital, Taipei, 11217 Taiwan; 6 Division of Transfusion Medicine, Department of Medicine, Taipei Veterans General Hospital, Taipei, 11217 Taiwan; 7 Comprehensive Breast Health Center, Taipei Veterans General Hospital, Taipei, 11217 Taiwan; 8 Faculty of Medicine, National Yang Ming University, Taipei, 112 Taiwan; 9 Department of Biochemistry, College of Medicine, Kaohsiung Medical University, Kaohsiung, 80708 Taiwan; 10 The Ph.D.Program for Translational Medicine, College of Medical Science and Technology, Taipei Medical University, Taipei, 11031 Taiwan

**Keywords:** TAOK3, NF-κB, Breast cancer, Anti-microtubule drug resistance

## Abstract

**Background:**

Chemotherapy is currently one of the most effective treatments for advanced breast cancer. Anti-microtubule agents, including taxanes, eribulin and vinca-alkaloids are one of the primary major anti-breast cancer chemotherapies; however, chemoresistance remains a problem that is difficult to solve. We aimed to discover novel candidate protein targets to combat chemoresistance in breast cancer.

**Methods:**

A lentiviral shRNA-based high-throughput screening platform was designed and developed to screen the global kinome to find new therapeutic targets in paclitaxel-resistant breast cancer cells. The phenotypes were confirmed with alternative expression in vitro and in vivo. Molecular mechanisms were investigated using global phosphoprotein arrays and expression microarrays. Global microarray analysis was performed to determine TAOK3 and genes that induced paclitaxel resistance.

**Results:**

A serine/threonine kinase gene, *TAOK3*, was identified from 724 screened kinase genes. TAOK3 shRNA exhibited the most significant reduction in IC50 values in response to paclitaxel treatment. Ectopic downregulation of TAOK3 resulted in paclitaxel-resistant breast cancer cells sensitize to paclitaxel treatment in vitro and in vivo. The expression of TAOK3 also was correlated to sensitivity to two other anti-microtubule drugs, eribulin and vinorelbine. Our TAOK3-modulated microarray analysis indicated that NF-κB signaling played a major upstream regulation role. TAOK3 inhibitor, CP43, and shRNA of NF-κB both reduced the paclitaxel resistance in TAOK3 overexpressed cells. In clinical microarray databases, high TAOK3 expressed breast cancer patients had poorer prognoses after adjuvant chemotherapy.

**Conclusions:**

Here we identified TAOK3 overexpression increased anti-microtubule drug resistance through upregulation of NF-κB signaling, which reduced cell death in breast cancer. Therefore, inhibition of the interaction between TAOK3 and NF-κB signaling may have therapeutic implications for breast cancer patients treated with anti-microtubule drugs.

**Video abstract**

## Introduction

Breast cancer is currently the most common cancer among women worldwide as well as the third leading cause of cancer deaths in the United States [1]. For locally advanced breast cancer, either paclitaxel- or docetaxel-containing adjuvant regimens are commonly used in clinical practice, with significantly better prognoses than other cytotoxic agents [2]. A new generation of anti-microtubule inhibitors, such as eribulin, has also shown survival benefits in refractory metastatic breast cancer, which indicates the important role of microtubule-targeted drugs in preventing breast cancer recurrence and controlling progression [3, 4]. However, taxane resistance eventually developes in approximately 90% of patients despite excellent initial therapeutic efficacy [5]. Several mechanisms of taxane resistance has previously been described: overexpression of multidrug resistance protein (MDR) genes, class III β-tubulin, and epithelial-mesenchymal transition [6–10]. The development of new agents to overcome resistance to taxane or other microtubule-targeting drugs is, therefore, indispensable to the advancement of disease treatment [11].

A systemic biology approach to screening of drug sensitivity has been used for the past 10 years in cancer treatment [12–15]. In order to discover novel therapeutic agents, shRNA is particularly valuable in identifying the mechanism of action of a compound with new anticancer indications and identifying potential targets. Furthermore, a kinome-based shRNA library for screening kinase protein inhibitors is manageable; there are 724 kinase proteins, and most of them have been implicated in primary cancer processes such as activating metastasis, sustaining proliferation, resisting cell death, etc [16–18] Currently, more than 10,000 candidate compounds have been patented to inhibit kinase activation [19]. Some kinase inhibitors have been approved for anti-cancer therapies, for example, BCR–ABL inhibitors have been approved for chronic myeloid leukemia [20], BRAF inhibitors have been approved for melanoma [21] and HER2 (also known as ERBB2) inhibitors have been approved breast cancer [22]. Other kinase inhibitors, such as Janus kinase (JAK) inhibitor tofacitinib, have been approved by FDA for the treatment of rheumatoid arthritis and could potentially be repurposed as novel anticancer agents.

In the present study, we utilized 724 shRNAs to generate a kinase library that we used to screen for novel genes responsible for taxane resistance in breast cancer. Knock down of a top ranking gene, *TAOK3*, overcome taxane resistance in breast cancer both in vitro and in vivo. Further pathway analysis revealed that TAOK3 may activate NF-κB signaling and blocking TAOK3-NF-κB signaling reduced the paclitaxel-resistance. Furthermore, clinical data were correlated with poor prognosis in breast cancer patients with high TAOK3 expression who accepted adjuvant therapy. This study reveals a new potential target for anticancer kinase inhibitors and future breast cancer treatments.

## Methods

### Cell lines and media

The 293 T, BT-20, BT-483, Hs578T, and MB 157 cell lines were cultured in Dulbecco’s modified Eagle’s medium (DMEM, Gibco, USA) supplemented with 10 mM L-glutamine and 10% fetal bovine serum. The AU565, Hcc38, Hcc70, Hcc1143, Hcc1937, Hcc1806, and T-47D cell lines were cultured in RPMI-1640 (Gibco, USA) medium supplemented with 10 mM L-glutamine and 10% fetal bovine serum. SKBR3 cells were grown in McCoy’s 5A medium (Gibco, USA) supplemented with 10 mM L-glutamine and 10% fetal bovine serum. MCF-7 cells were grown in MEM with 10% fetal bovine serum, 0.01 mg/mL human recombinant insulin and 10 mM L-glutamine. The cells were maintained in an incubator at 37 °C in 5% CO_2_. MDA-MB-231, MDA-MB-453, and MDA-MB-468 cells were cultured in L-15 medium supplemented with 10 mM L-glutamine and 10% fetal bovine serum. The cells were incubated in a humidified 37 °C incubator without extra CO_2_ supply. All cell lines were purchased from American Type Culture Collection (ATCC).

### shRNA lentivirus production on solid-phase transfection platform

pGIPZ-shRNA glycerol stocks were purchased from Open Biosystems. DNA extraction was performed in a 96-well plate using a semi-automated Biomek-FX liquid handler and a ChargeSwitch nucleic acid purification kit (Invitrogen, USA). The negative control, which contains the pGIPZ vector backbone with a portion of scrambled shRNA (non-silencing gene), was included in each coated plate. A total of 150 ng of pGIPZ-shRNA plasmid and lentiviral packaging plasmids were used for plate coating on 96-well plates. Arrest-In (600 ng) (Open Biosystems, USA) was used as a transfection reagent, and the DNA-arrest-in complex was plated before the addition of 25% gelatin (Sigma, USA) [23]. The plates with DNA complexes were dried at 65 °C for 90 min before storage at − 80 °C.

### Cell viability assay

Paclitaxel (Sigma Aldrich, USA) was dissolved in DMSO and diluted in the growth medium. Eribulin (Halaven®, NerPharMa S.r.l., Italy) and vinorelbine (Navelbine®, Pierre Fabre, France) were diluted in the growth medium. High throughput cell viability assays were performed on 384-well white plates using Cell Titer Glo (Promega, USA). For general cytotoxicity assays, 2000 cells were seeded into each well 24 h before drug treatment. AlamarBlue (Invitrogen, USA) was added at 1/10 the total volume in a well and incubated for 2–4 h. Fluorescence intensity was recorded using an ELISA reader (PerkinElmer, USA). A cell imaging device, IncuCyte (IncuCyte, USA), was used to determine the cell confluence on 96-well plates over time.

### Transfection, transduction, and cDNA cloning

All transfections for high-throughput screening were performed in DNA-coated 96-well plates with 50,000,293 T cells per well. The viral supernatants were collected at 48 and 72 h post transfection and stored at − 80 °C. Breast cancer cell line Au565 was transduced using the virus collected from transfection in 293 T cells. Transduction for screening was performed in 384-well plates with 8 μg/mL of polybrene (Sigma Aldrich, USA). Stable breast cancer cell lines with targeted shRNA and cDNA were generated with 10 μg/mL puromycin and blasticidin, respectively (InvivoGen, Hong Kong). *TAOK3* cDNA was cloned from an ORF clone and sub-cloned into pLenti6.3 Gateway vector using Gateway cloning systems according to the manufacturer’s protocol (Invitrogen, USA).

### RNA extraction and real-time quantitative PCR

Total RNA was extracted using Tri-reagent (Invitrogen, USA) and chloroform. The cDNA was synthesized by reverse transcriptase (Stratagene, USA) at 42 °C. Real-time PCR was performed using SyBr Green (Fermentas, Canada) and specific TAOK3 primers (5’gtgggcacaccttactggat3’ and 5’aacgttggggagtcattctg3’). Real-time PCR was performed in a BioRad 96-well real-time PCR detection system.

### Microarray analysis

Total RNA was extracted with the RNeasy Mini kit (Qiagen, USA) and qualified with a Bioanalyzer (Agilent Technologies, USA). All samples were analyzed using Affymetrix GeneChip Human Genome U133 plus 2.0 arrays according to the manufacturer’s instructions. The data were normalized and analyzed by GeneSpring software (Agilent Tech., USA). Genes that changed more than threshold (1.5- and 2-fold) were sorted and further submitted to a computational simulation using Ingenuity Pathway Analysis (IPA, QIAGEN, USA) online tools to predict potential upstream regulators and the canonical pathways (pathways that represent common properties of a particular signaling module).

### Protein extraction and Western blotting

Protein was extracted using RIPA buffer (20 mM Tris-HCl at pH 7.4, 150 mM NaCl, 0.5% Nonidet P-40, 1 mM EDTA, 50 μg/mL leupeptin, 30 μg/mL aprotinin, and 1 mM phenylmethylsulfonyl fluoride) containing proteinase inhibitors. Protein concentration was determined with the BCA kit (Thermo Scientific, Rockford, USA) using BSA as the standard. Approximately 20–100 μg of protein was loaded in an SDS-PAGE (TRIS-based), and blotting was performed on a nitrocellulose membrane (Amersham, Arlington Heights, IL, USA). Antibodies against TAOK3 (1:1000, #10158–2-AP, Proteintech, USA), phospho-p38 (1:1000, #4511, Cell signaling Tech.), p38 (1:2000, #9212, Cell Signaling Tech.), phospho-p65 (1:2000, #3033, Cell Signaling Tech.), p65 (1/2000, #4764, Cell Signaling Tech.), phospho-p53 (1:1000, #2521, Cell Signaling Tech.), p53 (1:500, #sc-126, Santa Cruz), caspase-3, (1:1000, #9662, Cell Signaling Tech.), PARP, (1:1000, #9542, Cell Signaling Tech.), β-actin (1:10000, Sigma) and α-tubulin (1:10000, Sigma) were diluted in blocking buffer. A secondary anti-mouse or anti-rabbit antibodies conjugated with HRP (Jackson ImmunoResearch Lab., USA) was used with 1:5000 dilution in blocking buffer. Visualization of the western blots was performed using the ECL Pro set (PerkinElmer) and X-ray radiography.

### Caspase assay

Caspase assays were performed on white 96-well plates according to the manufacturer’s protocol using caspase-3 Glo (Promega, USA). Approximately 20,000 cells were seeded onto the 96-well plate, and paclitaxel was added to the cells at 24 h before the caspase assay. The luciferase activity was measured using a Victor3 photometer, and the relative caspase activity was normalized with the corresponding AlamarBlue values.

### TUNEL assay

Tissue slides were dewaxed felled by the detection of DNA cleavages using fluorescein-dUTP labeling with the enzyme terminal deoxynucleotidyl transferase (TdT) (In situ cell death detection kit, fluorescein, Roche, Switzerland). After labeling, slides were covered with mounting medium and sealed with nail polish. The images were scanned by the fluorescent slide scanner (Scanscope FL, Aperio, USA).

### Promoter assay

pGL4-NF-κB response vectors (Promega, USA) were transfected into cells for 48 h. The promoter activity was determined with 0.15 mg/mL luciferin and recorded by an IVIS Spectrum (PerkinElmer, USA). The activity values were normalized to the fluorescence intensity of AlamarBlue (Invitrogen, USA).

### Xenograft mouse model

All animal experiments were approved by the Academia Sinica Institutional Animal Care and Unitization Committee (14–03-665). Immunodeficient (NOD-SCID) female mice (6–8 weeks old) were used. A total of 5 × 10^6^ cells were re-suspended in 0.1 mL of PBS and injected subcutaneously. Tumor sizes were measured weekly and volume was calculated by 1/2 ab^2^ mm^3^. Taxol (paclitaxel, Bristol-Myers Squibb) in 0.2 mL PBS was administered via tail vein injection twice weekly. The Hs578T group was treated at approximately 6 mg/kg, the Hcc1806 high dose group was treated at approximately 3 mg/kg and the low dose group was treated at approximately 1 mg/kg.

### Statistical analysis

All statistical calculations were performed in Excel, and the results are shown as the means ± standard deviations. The non-silencing control was used as the internal reference on each plate. Statistical significance was performed using a 2-samples t-test and a one-way ANOVA with a *p*-value less than 0.05.

## Results

### Identification of paclitaxel-resistant gene targets in breast cancer cells

**Fig. 1:**
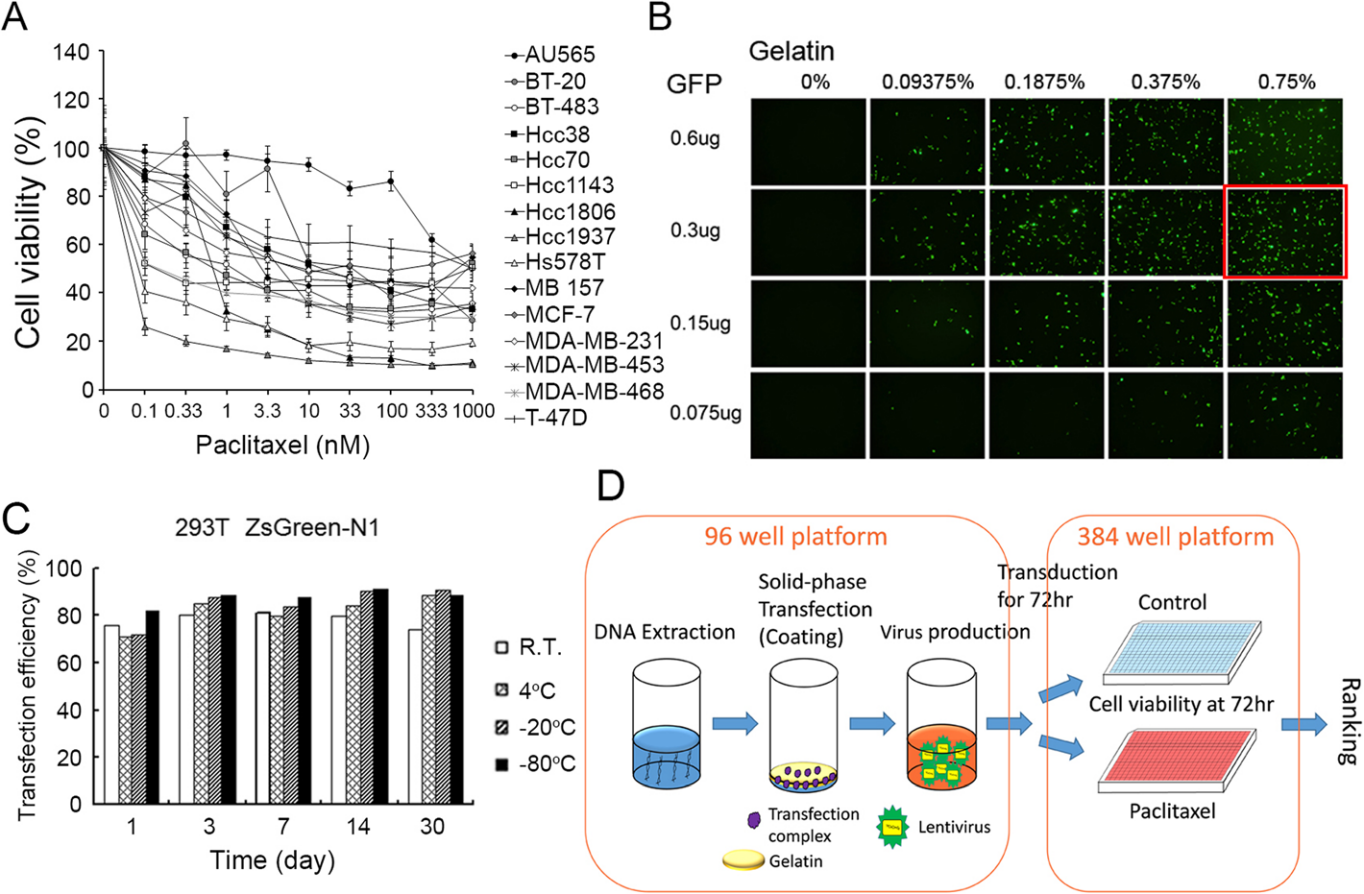
Preparation for shRNA screening platform. **a** The cell toxicity assay of paclitaxel in variant breast cancer cell lines. **b** The GFP transfection efficiency of different coating conditions. **c**. The stability of coating plates after storing in variant conditions. **d** Diagram of shRNA screening procedure of breast cancer cells

**Fig. 2:**
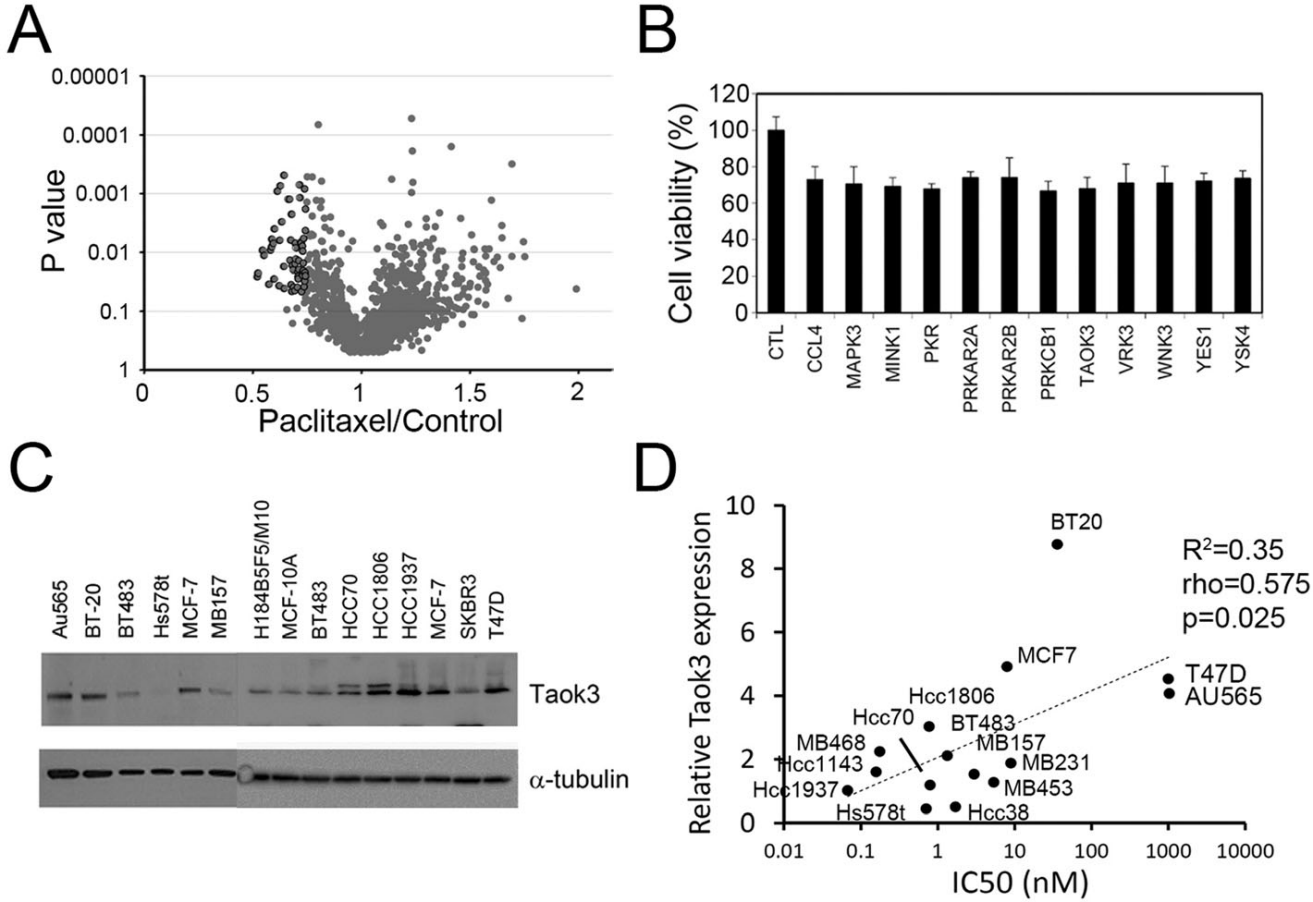
Identification of TAOK3 with kinome shRNA screening in breast cancer cell lines. **a** The distribution of whole kinome shRNA screening clones of “*p*-value” and “paclitaxel/control ratio”. Black circles indicate the candidates with > 25% inhibition and *p*-value < 0.05. **b** The second round of the cell toxicity assay of paclitaxel with candidate shRNA treatment. **c** TAOK3 protein expression of breast cancer cell lines. **d** Correlation plot between IC50 of paclitaxel and the quantitative protein expression in each of breast cancer cell lines

After evaluating the IC50 of paclitaxel in 15 breast cancer cell lines, we found that Au565 was the most paclitaxel-resistant cell line to paclitaxel (Fig. 1a, Table S1). For the screening method, we used a solid phase transfection system to produce shRNA lentivirus in 96 well plate. First, we determined the optimal conditions to achieve the highest transfection efficiency (Fig. 1b). We also evaluated the stability of the transfected, coating plates at different temperatures and time points. We found that 3 day of plate coating was optimal and that the efficiency was maintained for more than 30 days in the refrigerator (Fig. 1c). Next, we used 293 T cells for high-throughput lentiviral screening. In the screening, we prepared the shRNA lentivirus in 96-well plates containing 724 kinase genes; altogether, we created 1646 shRNA clones (Fig. 1d). We compare the results of the “before” and “after” paclitaxel treatment groups (the altered ratio distributions are shown in Fig. 2a). We selected the top 50 candidate genes whose knockdown induced paclitaxel sensitivities (listed in Table S2). Many of the identified kinases belonged to the MAPK, PI3K-AKT, or NF-κB signaling pathway. We selected some novel kinase genes for second round validation, and our results showed that the knockdown of TAOK3 created the highest sensitivity to paclitaxel (Fig. 2b). We found the TAOK3 protein expression level was positive correlated to paclitaxel IC50 values in breast cancer cell lines (Fig. 2c and d). Taken together, these facts indicate that TAOK3 may be associated with paclitaxel resistance.

### Knockdown of TAOK3 increased chemosensitivity in breast cancer cells

**Fig. 3:**
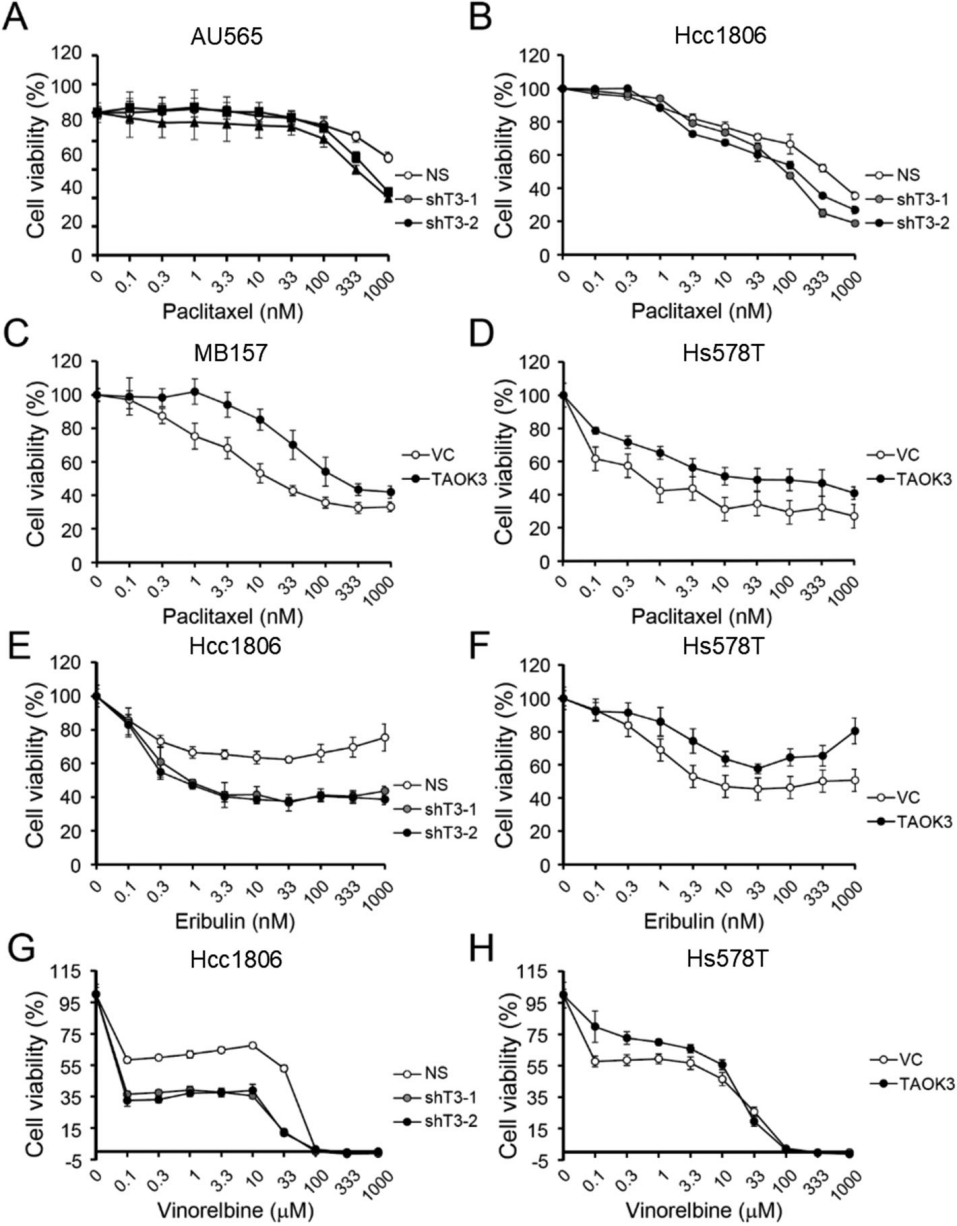
The cytotoxicity effects of anti-microtubule drugs in TAOK3-modulated breast cancer cell lines. **a** The cytotoxicity assay of paclitaxel in AU565 with TAOK3 shRNAs and control. **b** The cytotoxicity assay of paclitaxel in Hcc1806 with TAOK3 shRNAs and control. **c** The cytotoxicity assay of paclitaxel in MB157 with TAOK3 overexpressed and control. **d** The cytotoxicity assay of paclitaxel in Hs578T with TAOK3 overexpressed and control. **e** The cytotoxicity assay of eribulin in Hcc1806 with TAOK3 shRNAs and control. **f** The cytotoxicity assay of eribulin in Hs578T with TAOK3 overexpressed. **g** The cytotoxicity assay of vinorelbine in Hcc1806 with TAOK3 shRNAs and control. **h** The cytotoxicity assay of vinorelbine in Hs578T with TAOK3 overexpressed

First, we determined the endogenous expression levels of TAOK3 in 12 breast cancer cell lines. The expression of TAOK3 in normal breast epithelial cells (H184B5F5/M10 and MCF-10A) was significantly lower than in breast cancer cells (Fig. 2c). To determine the effect of TAOK3 on paclitaxel sensitivity, we knocked down the TAOK3 expression in the high expression cell line, AU565, Hcc1806, and, SKBR3, with two shRNA clones (Figure S1A). Cells with TAOK3 shRNA treatment showed enhanced sensitivity to paclitaxel (Fig. 3a and b). SKBR3 cells, which had moderate TAOK3 expression, had an increased enhancement of paclitaxel sensitivity (Figure S1B). In addition, we overexpressed TAOK3 in the low endogenous TAOK3 cells, Hs578T and MB157. The overexpression of TAOK3 conferred the sensitive breast cancer cells with an increased resistance to paclitaxel (Fig. 3c and d). The overexpression of TAOK3 increased the original IC50 of paclitaxel by 8.9-fold and 11.1-fold in Hs578T and MB157 cells, respectively. Next, we treated cells with the anti-microtubule drugs eribulin and vinorelbine [24]. Downregulated TAOK3 expression in breast cancer cells enhanced the response to the drugs in non-silenced cells (Fig. 3e and g). In TAOK3 overexpression cells, cells exhibited higher drug resistance than the control group (Fig. 3f and h). However, these changes in drug response were not observed with the drugs cisplatin and doxorubicin, which interact with DNA (Figure S2). The chemoresistance effect of TAOK3 was specific to microtubule-targeted drugs. Notably, the TAOK3-related growth inhibition or enhancement was paclitaxel-dependent, as no significant difference in cell growth were observed in cells with either TAOK3 shRNA or cDNA when compared to the control cells (Figure S3).

### TAOK3 knockdown increased taxane-induced cell apoptosis

**Fig. 4:**
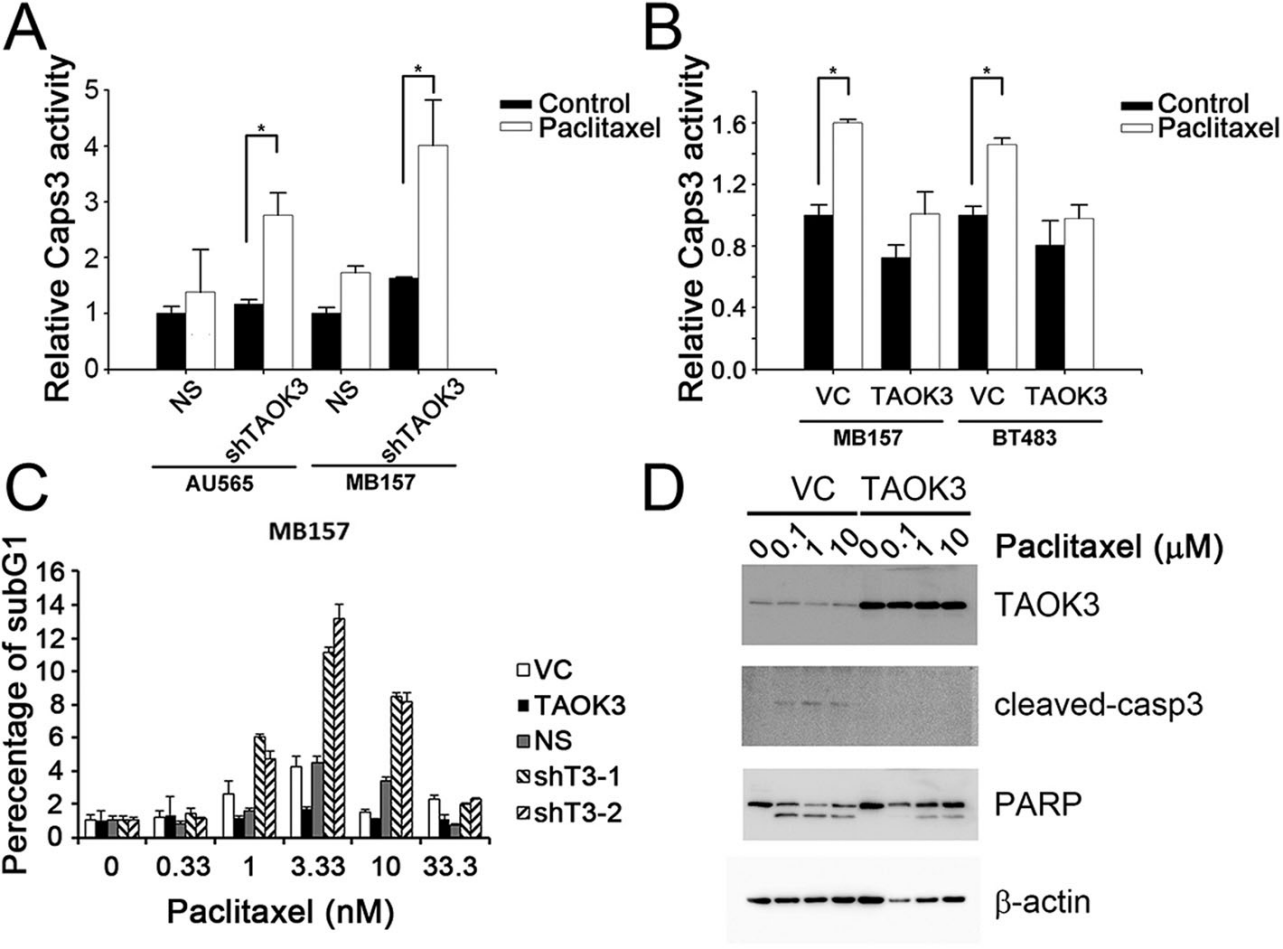
TAOK3 expression altered cell death in taxane-treated cells. **a**. Caspase-3 activity was measured at 24 h after paclitaxel treatment using Caspase-Glo 3/7 assay in AU565 and MB157 cells with TAOK3 knockdown. The relative caspase activity was calculated using the bioluminescence value divided by the value of cell viability reading from AlamarBlue. **b** The relative caspase-3 activity in TAOK3-modified MB157 and BT483 cells treated with paclitaxel. * indicates *p*-values < 0.05. **c** The distribution of sub-G1 percentage in TAOK3-modified MB157 cells treated with paclitaxel for 24 h. The TAOK3 expression panel was detected by western blotting. **d** Expression of TAOK3, cleaved caspase-3, and PARP in Hs578T cells treated by paclitaxel for 24 h

To understand the mechanism of TAOK3-dependent drug resistance, we measured the cell death by detecting caspase-3 activity, sub-G1 percentage, and PARP cleavage after treatment with paclitaxel in TAOK3-manipulated cells. The results showed that caspase-3/7 was prominently activated upon treatment with an IC50 dose of paclitaxel in AU565 and MB157 cells after the depletion of TAOK3 (Fig. 4a). There were no significant differences in caspase-3/7 activation observed at 24 h in control cells treated with paclitaxel or in untreated cells. Conversely, a significant difference was observed in shTAOK3-modified cell lines treated with paclitaxel. The overexpression of TAOK3 conferred MB157 and BT483 cells with more resistance to paclitaxel treatment, and no significant difference in caspase-3/7 activity was observed in the TAOK3-overexpressed cells (Fig. 4b). For the cell cycle analysis, we harvested MB157 cells after treating with 0, 0.33, 1, 3.33, 10 and 33.33 nM paclitaxel for 24 h. At 3.33 nM treatment, the highest sub-G1 percentage was observed in the TAOK3 knockdown groups. In contrast, the sub-G1 percentage in the cell population with TAOK3 overexpressed did not dramatically increased after treatment with paclitaxel (Fig. 4c). However, if the dose of paclitaxel was too high, the sub-G1 percentage decreased the cells became arrested at the G2 phase. We also determined the cleavage of caspase-3 and PARP in apoptotic Hs578T cells with TAOK3 overexpression compared to the control with 0.1, 1 and 10 μM paclitaxel treatment after 24 h incubation. The results showed that TAOK3 overexpression reduced the cleavage of both caspase-3 and PARP (Fig. 4d).

### Overexpression of TAOK3 increased chemoresistance in breast cancer cells in vivo

**Fig. 5:**
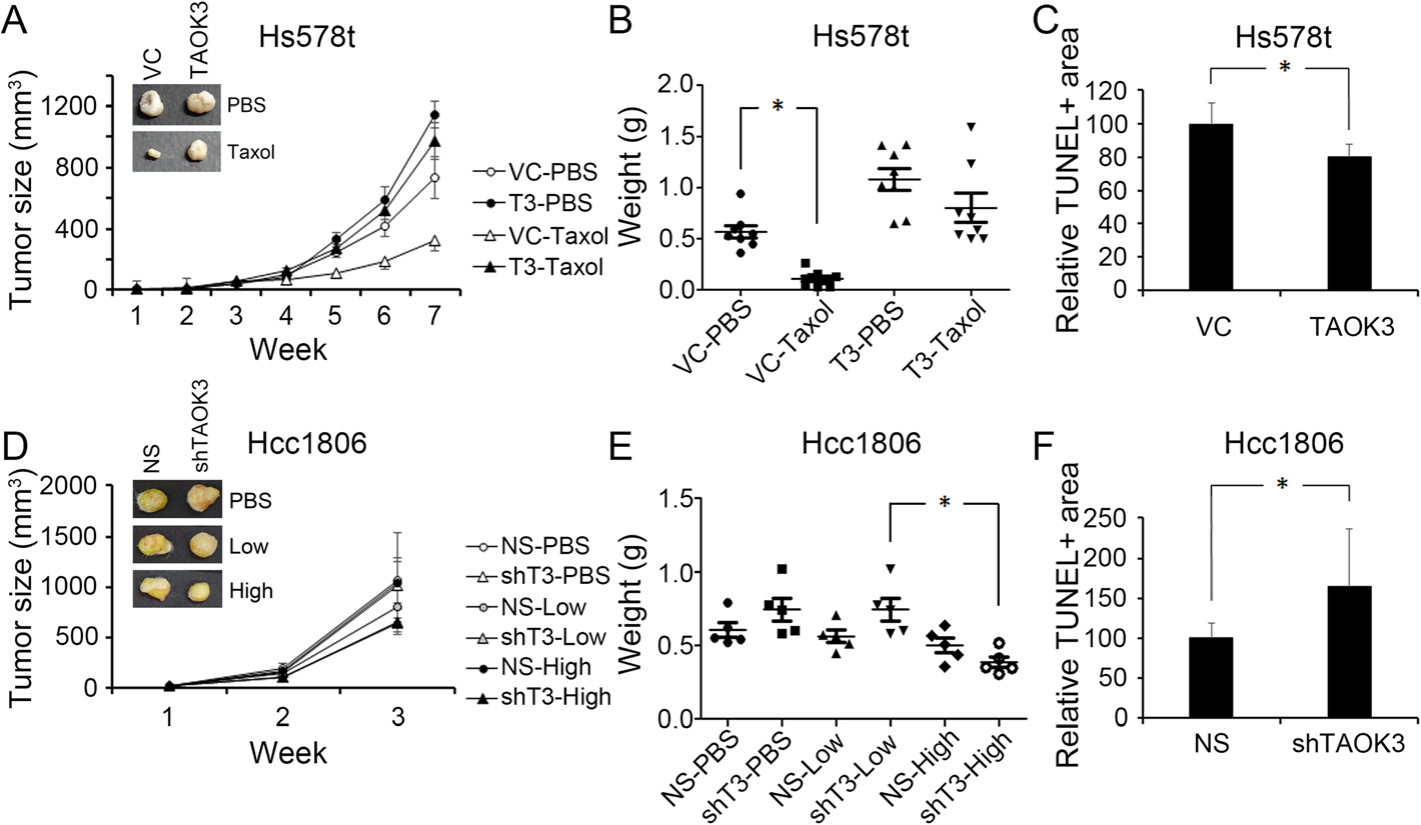
Effects of the production of TAOK3 on tumors growth and death after paclitaxel treatments. **a** The tumor growth curve of Hs578T-VC and Hs578T-TAOK3 with/without paclitaxel (6 mg/kg). Tumor size was calculated by 1/2ab^2^. **b** Tumor weight distribution among the four indicative groups. * indicates *p* < 0.05. **c** Fluorescence photography of TUNEL stain in Hs578T-VC and Hs578T-TAOK3 cells after treating paclitaxel. **d** The tumor growth curve of Hcc1806-NS and Hcc1806-shTAOK3 with/without paclitaxel treatment. The treatment was initiated at week 2. “Low dose” refers to 1.1 mg/kg and “high dose” refers to 3.4 mg/kg paclitaxel in each injection. **e** Tumor weight distribution of the six indicative groups of Hcc1806. * indicates *p* < 0.05. **f** Fluorescence photography of TUNEL staining in Hcc1806-NS and Hcc1806-shTAOK3 cells after treatment with paclitaxel

To access the effect of paclitaxel treatment on different TAOK3 expression levels in vivo, we determined the paclitaxel response effects in Hcc1806 and Hs578T using an in vivo subcutaneous xenograft tumor model. The experimental mice were injected with the cells at two sides: one side was injected with control cells and the other side was injected with the TAKO3-overexpressed clone. When the tumor sizes reached approximately 0.5 cm in diameter, paclitaxel (TAXOL® reagent) was intravenously injected into mice. The experimental mice received the drug twice a week until the tumors were bigger than 1500 mm^3^. Without paclitaxel treatment, there was no significant difference between the control and TAOK3-overexpressed groups. However, the paclitaxel treatment significantly inhibited the tumor growth of control cells (73%), compared to the TAOK3-overexpressed group (17%) at week 7 (Fig. 5a). A comparison of the tumor weight between the 4 groups revealed significant inhibition after paclitaxel treatment in the control group (Fig. 5b). In addition, tumor sections of similar size were selected for TUNEL staining to evaluate cell death after paclitaxel treatment. The results showed that the paclitaxel-treated group had a smaller percentage of positive staining in non-necrosis regions of Hs578T-TAOK3 tumor than Hs578T-VC tumor (Figs. 5c, S4). In the Hcc1806-shTAOK3 groups, the data showed dose-dependent enhanced paclitaxel sensitivity (Fig. 5d). High doses of paclitaxel decreased tumor weights more significantly than low dose (shown in Fig. 5e). The TUNEL staining also revealed that cell death was higher in the Hcc1806-shTAOK3 tumors than in the Hcc1806-NS tumors. (Figs. 5f, S4).

### TAOK3 regulated paclitaxel resistance via the NF-κB/PTGS2 signaling pathway

**Fig. 6:**
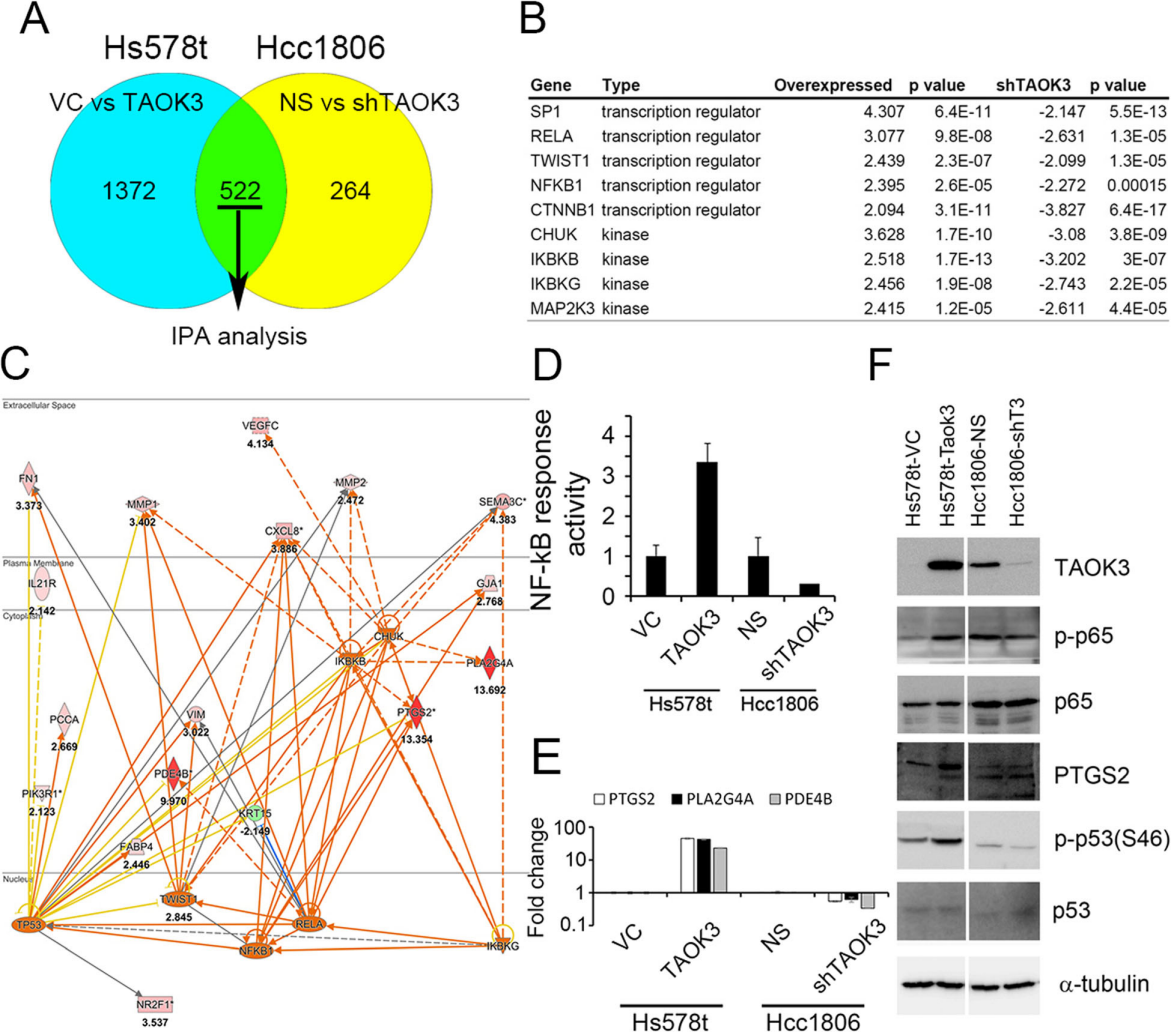
The discovery and evaluation of TAOK3-associated pathway analysis. **a** The diagram of intersecting genes between upregulated and downregulated TAOK3 datasets. **b** The top-ranking kinase and transcription factor list of upstream analysis. **c** IPA pathway network of RELA, NF-κB and p53 and the downstream genes with more than two-fold differences. **d** Determination of the endogenous activity of NF-κB by a NF-κB response assay in alternative TAOK3 cell lines. **e** Determination of the RNA expression of PTGS2, PLA2G4A and PDE4B by realtime PCR. **f** Determination of the endogenous protein changes after upregulated/downregulated TAOK3 in cell lines

We performed RNA microarray analysis to analyze 522 genes from Hs578T cells with TAOK3 overexpression and Hcc1806 cells with TAOK3 knockdown (Fig. 6a). After the IPA upstream analysis, both sets of cells showed enrichment of SP1, TWIST1, CHUK and NF-κB signaling, indicating that these pathways are involved in upstream regulatory functions (Fig. 6b). We also stained a phosphoprotein array to determine the changes of various phosphokinase (Figure S5A). After semi-quantitation, phosphorylation of p53 showed the largest enhanced in overexpression condition (Figure S5B). However, NF-κB-associated kinases were not presented in the phosphoprotein array. Genes associated with RELA, NF-κB and p53 pathways and genes that changed more than 2-fold in both cell sets were showed in Fig. 6c (Figure S4C and D). In this signaling pathway, we found that PLA2G4A, PTGS2, and PDE4B (shown as red diamonds) exhibited at least 10 times increase with TAOK3 overexpression. We confirmed NF-κB activity in these cells by using a NF-κB promoter assay. Our result showed that the expression of TAOK3 and NF-κB activity were positively correlated (shown in Fig. 6d). The RNA expression levels of PTGS2, PLA2G4A, and PDE4B were determined with real-time PCR (Fig. 6e). PTGS2 is known as an NF-κB-directed target. These phenotypes were validated with an analysis of endogenous protein expression (Fig. 6e). Phospho-p65 was found to be upregulated in TAOK3-overexpressed cells; however, it was undetectable in Hcc1806-NS and Hcc1806-shTAOK3 cells. The results of the PTGS2 analysis similar to the p-p65 results. Phospho-p53 was found to be upregulated and downregulated in TAOK3-modified cells. Hcc1806 had two-base-pair insertion at codon 256; thus, the protein was the wrong molecular weight, but phospho-p53 was found with a very low expression level. In addition, a mutant p53 protein (V157F) was also found in Hs578T cells. Furthermore, other cells such as AU545 (wildtype p53), SKBR3 (L175H), and MB157 (p53, del 26 bp at 261) exhibited responses to paclitaxel. These results indicate that the interaction with TAOK3 and p53 may not be critical to the cell death induced by paclitaxel. Taken together, we conclude that TAOK3 reduced paclitaxel cytotoxicity through the activity of the NF-κB signaling pathway.

### Reduce paclitaxel resistance with a TAOK3 inhibitor and NF-κB shRNA in TAOK3-overexpressed cells

**Fig. 7:**
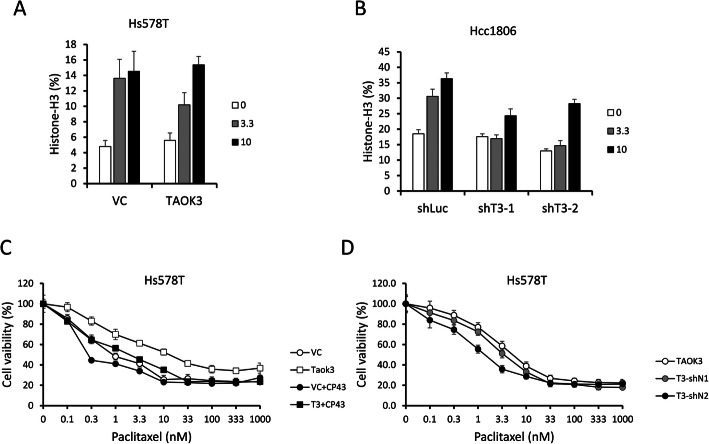
Effects of CP43 and NF-κB shRNA in TAOK3-modulated cells. **a** The percentage of mitotic cells after CP43 treatment for 24 h in Hs578T-VC and Hs578T-TAOK3 cells. The cells were stained with FITC conjugated Histone H3-S10p antibody and PI. **b** The percentage of mitotic cells after CP43 treatment for 24 h in Hcc1806-shLuc, Hcc1806-shTAOK3–1 and Hcc1806-shTAOK3–2. **c** The cytotoxicity assay of paclitaxel in Hs578T cells with TAOK3 overexpressed and control (white icon). The combination of paclitaxel and CP43 (3.3 μM) is shown by a black icon. **d** The cytotoxicity of paclitaxel in Hs578T-TAOK3-overexpressed cells with NF-κB shRNAs and control

To overcome the TAOK3 effects on paclitaxel resistance, we tested a specific inhibitor of the TAOK3 family, CP43 [25], and used NF-κB shRNA on a TAOK3 overexpression clone. In Hs578T cells, we found that CP43 increased the percentage of mitotic cells (histone H3-pS10-positive) in all clones; however, in TAOK3-overexpressed cells, the percentage increase was dose-dependent (Fig. 7a). When we knocked down TAOK3, the response to CP43 were reduced (Fig. 7b). We also found that when we combined CP43 and paclitaxel, CP43 reduced paclitaxel resistance in TAOK3-overexpressed cells but only slightly reduced paclitaxel resistance in vector control (Fig. 7c). We further knocked down NF-κB in Hs578T cells, and we did not observe a dramatic change in mitotic cells (Figure S6A). We also did not observe the synergistic effects of NF-κB shRNA and paclitaxel in the vector control group (Figure S6B). Conversely, we did notice a reduction in paclitaxel resistance after NF-κB shRNA knockdown in the TOAK3-overexpressed cells (Fig. 7d, clone T3-shN2). Such results may indicate that inhibition of TAOK3-NF-κB signaling is a potential treatment on reducing paclitaxel-resistance in breast cancer cells.

### TAOK3 overexpression correlated to poor prognosis of breast cancer patients who accepted adjuvant chemotherapy

**Fig. 8:**
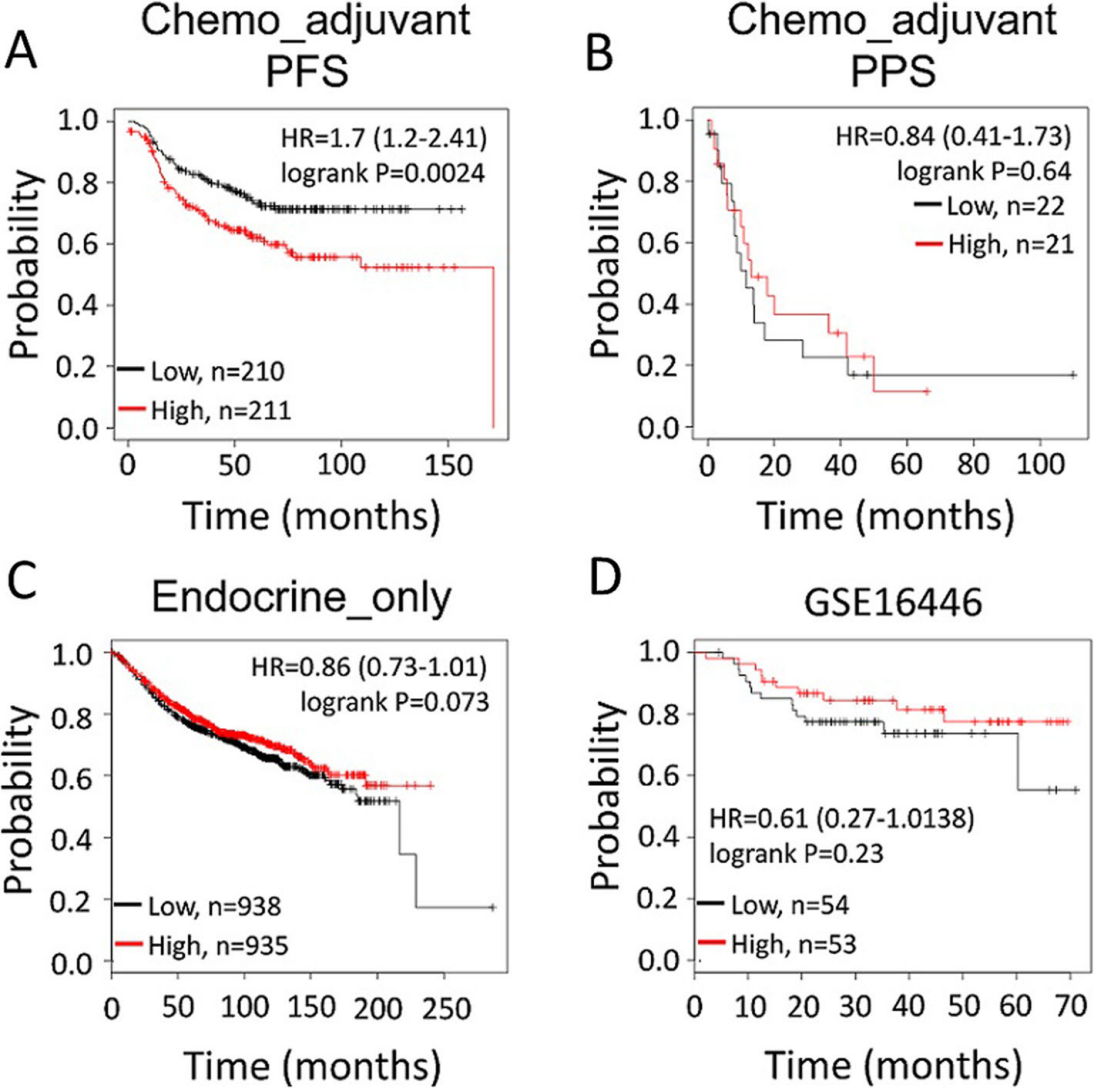
Kaplan–Meier plots of TAOK3 in different sub-cohorts of clinical breast cancer patients. **a** The breast cancer patients with only systemic adjuvant chemotherapy (*n* = 421). **b** The same population as Fig. 7a but with post progression survival (*n* = 43). **c** The patients only with adjuvant endocrine therapy (*n* = 1873). **d** The patients who accepted epirubicin monotherapy as neoadjuvant treatment for breast cancer (*n* = 107)

Using the bioinformatics tool “Kaplan-Meier Plotter (https://kmplot.com/analysis/)” [26], which contains both publicly available array profiles and clinical data, we evaluated whether TAOK3 was associated with the prognoses for breast cancer patients. Because taxane-based adjuvant chemotherapy is used in most cases of high-risk breast cancer, we focused on patients with subsequent adjuvant chemotherapy (except endocrine therapy); we found that higher TAOK3 expression was significantly correlated to poor recurrence-free survival (HR = 1.7(1.2–2.41), *p* = 0.0024) (Fig. 8a) rather than post progression survival (Fig. 8b). This difference was not observed in patients who accepted endocrine-only adjuvant treatments (Fig. 8c). We further investigated possible correlation of TAOK3 with other chemotherapy drugs. Using the GSE16446 database, which contains data about breast cancer patients who accepted only epirubicin as neoadjuvant therapy, we found no significant difference in recurrence-free survival between high vs. low TAOK3-expressed patients (HR = 0.61(0.27–1.38), *p* = 0.23) (Fig. 8d). The above results provide more evidence that TAOK3 is associated with breast cancer prognosis specifically after taxane treatment.

## Discussion

In this study, we established a simple and stable solid-phase transfection method to systematically produce many different shRNA lentiviruses. We prepared a kinase library contained 724 genes to screen for paclitaxel-resistance genes in the most resistant breast cancer cell line. The top 50 candidate genes included many genes related to paclitaxel resistance, such as AKT1, SRC, etc., but to date, many have not been mentioned in the literature [27, 28]. After a similarity score analysis (score > 0.5) using the DAVID bioinformatics database [29], we found two major subsets in seven related clusters. One group included ABL2, YES1, BMX, and LCK, which have been associated with paclitaxel resistance [30–33]. The other group included MINK1, TSSK2, MARK1, MARK2, WNK3, VRK3, and TAOK3; however, this group has rarely been mentioned. The candidates that are showed in Fig. 2b are the genes that have rarely been reported to be involved in taxane resistance. TAOK3 shRNA initiated the most significant decline of paclitaxel IC50 values. We also found that the expression of TAOK3 in breast cancer cell lines was generally higher than that in normal cells (Fig. 2c). The expression of TAOK3 was positive correlative to the IC50 of paclitaxel in breast cancer cell lines (Fig. 2d). This phenomenon was also observed when different sensitivity to paclitaxel were caused by adjusting the expression of TAOK3 in different cell lines (Fig. 3a-d). In addition, we also found that TAOK3 expression was related to other anti-microtubule agents but relatively unlinked to the action of DNA damage agents (Figs. 3e-h, S2). The overexpression of TAOK3 rendered cells less likely to die due to paclitaxel, and in vivo experiments showed a similar pattern. In addition, we also found that the necrosis area was decreased when tumor with TAOK3 overexpression but without paclitaxel treatment (Figure S7). In vitro TAOK3 overexpression reduced the cleavage of caspase-3 and decreased DNA breakdown induced by paclitaxel treatment in vivo*.*

TAOK3 (thousand and one kinase 3) belongs to the STE20 family of serine/threonine kinase. Unlike other STE20-like kinase that indirectly enhanced the activity of JNK, ERK, and p38MAPK signaling, TAOK3 inhibited the activity of JNK but does not affect other MAPK signaling [34–37]. There are three known TAO kinases: TAOK1 ~ 3, which are activated by stress; for example, TAOK2 was found to render cells resistant to irradiation by enhancing the capability of initiating DNA damage-induced G2/M arrest [38]. TAOK3 was also found to directly phosphorylated the LATS1/2 and MST1/2 and act as a negative regulator to the effectors YAP/TAZ in the Hippo pathway [39]. Paclitaxel sensitivity has been highly correlated to ERK and p38 MAPK signaling [40, 41]. However, the modulation of TOAK3 expression did not affect JNK, ERK and p38 MAPK signaling in our TAOK3 overexpression kinase protein array. The microarray analysis indicated complex downstream signaling interactions of RELA and NFKB1 that induced the phosphorylation of MAPK, NF-κB and PI3K-AKT signaling pathways, which are involved in the paclitaxel response [42–44]. These signaling pathways enhance cell survival ability and regulates the anti-apoptosis proteins. Our data shown that NF-κB signaling was the most upregulated. The activation of NF-κB signaling has been reported to play a critical role in paclitaxel resistance in ovarian, non-small cell lung cancer, and breast cancer cells [45–47]. In our study, the overexpression of TAOK3 increased phosphorylation of NF-κB. The NF-κB signaling has been found to participate in crosstalk with other signaling pathways including AKT and p53 [48–50]. One study on the interaction between YAP and NF-κB has suggested verteporfin as a potential compound to reverse paclitaxel resistance in lung cancer [51]. Currently, there is no evidence that TAOK3 directly binds and activates the NF-κB protein; therefore, indirect modulation is more plausible. The links between TAOK3 and NF-κB still need to be explored.

Mitotic slippage is one of the primary mechanisms of action to paclitaxel [52]. Initially, mitotic arrest protects cells from chromosome segregation and generation of aneuploid cells. Normally, cells that slip out of mitosis stop dividing, become senescent or die [53]. However, some resistant cells can withstand mitotic catastrophe and survive. In a previous study, CP43 was found to inhibit TAOK1 and TAOK2, which caused an increase in the percentage of cells found in the mitotic phase via disrupting of the spindle assembly in nucleus [25]. In cell cycle analysis, this effect of CP43 is similar to the effect of paclitaxel. However, the effect of CP43 on TAOK3 is unknown. In our microarray analysis. TAOK3 modulation had no effect on the expression of TAOK1 or TAOK2 (Table S3). However, we observed that the number of cells in mitotic changed after CP43 treatment. Thus, TAOK3 may encourage mitotic slippage to reduce cell death or senescence; therefore, it would require higher CP43 to reach the threshold. More cells died when paclitaxel and CP43 were combined together (Fig. 7c). In another experiment, we used shRNA NF-κB to block TAOK3-NF-κB signaling in TAOK3-overexpressed cells. The effects were different than those of CP43. The number of cells in the mitotic phase was slightly reduced when NF-κB expression was knockdown. The synergistic effect with paclitaxel was shown in TAOK3-overexpressed cells but not in controls. This could be because NF-κB signaling was not critical to mitotic slippage but still played a role in paclitaxel resistance in the TAOK3-overexpressed cells.

Interestingly, we also found that TAOK3 is associated with chemosensitivity to other anti-microtubule agents such as eribulin and vinorelbine (Fig. 3e to h). Prior clinical trials have shown that eribulin is effective after prior exposure to taxanes [3, 54]. Moreover, vinorelbine is commonly used in chemotherapy after taxane exposure [55, 56]. Our findings warrant further investigation into taxane-exposed tumors and any correlation with the effects of subsequent treatment with eribulin or vinorebine. Our research suggests that TAOK3 may play a role in determining the chemo-sensitivity of tumors treated with anti-microtubule agents. However, more mechanistic studies are needed.

In summary, we found that TAOK3 expression enhanced the paclitaxel resistance of breast cancer cells via the NF-κB signaling pathway. However, the mechanism of TAOK3-NF-κB-PTGS2 pathway remains unclear. Although there are no known specific inhibitors of TAOK3, there are several commercial NF-κB inhibitors, and the inhibition of NF-κB signaling could provide a putative resolution for TAOK3-associated anti-microtubule drug resistance. In the future, TAOK3 as a molecular target in cancer treatment should be evaluated.

## Conclusions

In this study, we screened paclitaxel response-associated kinases and provided evidence that TAOK3 overexpression reduced the sensitivity to anti-microtubule drug in breast cancer cells and was correlated with poor outcomes in patients. TAOK3 to NF-κB signaling could be a new target for drug development and therapeutic strategies for breast cancer.

## Supplementary information

**Additional file 1: Supplement Table 1.** IC50 of paclitaxel among breast cancer cell lines. **Supplement Table 2.** Top 50 candidate list from kenome shRNA screening. **Supplement Table 3.** The fold change of TAOK family in microarray with TAOK3 modulation. **Supplement Figure 1.** Protein expression TAOK3 and cell viability of paclitaxel in SKBR3 (A) The effects of TAOK3 shRNA in Au565, Hcc1806 and SKBR3 and overexpressed TAOK3 in MB157 and Hs578t. (B) Paclitaxel sensitivity changes of shTAOK3 SKBR3 cells with paclitaxel treatment. **Supplement Figure 2.** The effect of cisplatin and doxorubicin with alternative TAOK3 expression. (A) Cell viability assay of cisplatin among Hcc1806-NS, Hcc1806-shTAOK3–1 and Hcc1806-shTAOK3–2. (B) Cell viability assay of cisplatin in Hs578t-VC and Hs578t-TAOK3. (C) Cell viability assay of doxorubicin among Hcc1806-NS, Hcc1806-shTAOK3–1 and Hcc1806-shTAOK3–2. (D) Cell viability assay of doxorubicin in Hs578t-VC and Hs578t-TAOK3. **Supplement Figure 3.** Growth effects of TAOK3 alternation. (A) Growth curve of shTAOK3 MB157 cells (B) Growth curve of TAOK3 overexpression MB157 cells. **Supplement Figure 4.** TUNEL staining in a similar size subcutaneous xenograft tumor of Hs578t-VC, Hs578t-TAOK3, Hcc1806-NS and Hcc1806-shTAOK3 with paclitaxel (Hs578t: 6 mg/kg and Hcc1806: 3 mg/kg) treatment for 24 h. **Supplement Figure 5.** Phosphokinase array and microarray analysis of TAOK3 affection. (A) The bot blot image of phosphoprotein array between Hs578t-VC and Hs578t-TAOK3. (B) Bar chart of top 10 increasing phosphorylated proteins. The semi-quantitation was measured with ImageJ.The network of intersection genes based on upstream analysis in (C) TAOK3 overexpression and (D) shRNA knockdown cells. The number showed the fold change of probe from microarray data. **Supplement Figure 6.** The effects of NF-κB shRNAs in Hs578T with TAOK3 modulation cells. (A) The mitotic percentage changes of NF-B shRNAs and control in Hs578T overexpressed and control cells. (B) The cytotoxicity of paclitaxel of NF-κB shRNAs and control in Hs578T control cells. **Supplement Figure 7.** IHC staining of TAOK3 in xenograft tumor. Cross-sections of alternative TAOK3 expression xenograft tumor without paclitaxel treatment with TAOK3 IHC staining.

## Data Availability

Clinical sample analysis was from the Kaplan-Meier Plotter database. Please refer the caption Fig. 8.

## Abbreviations

shRNA: Short hairpin RNA
siRNA: Small interfering RNA
TAOK3: TAO3 kinase
NF-κB: Nuclear factor kappa-light-chain-enhancer of activated B cells
MDR: Multidrug resistance protein
BCR: Breakpoint cluster region
ABL: Abelson murine leukemia viral oncogene
BRAF: B-Raf murine sarcoma viral oncogene homolog B
HER2: Human epidermal growth factor receptor 2
ERBB2: Erb-b2 receptor tyrosine kinase 2
ELISA: Enzyme-linked immunosorbent assay
cDNA: Complementary DNA
PCR: Polymerase chain reaction
IPA: Ingenuity pathway analysis
BCA: Bicinchoninic acid
HRP: Horseradish peroxidase
PARP: Poly (ADP-ribose) polymerase
ECL: Enhanced chemiluminescence
PBS: Phosphate-buffered saline
MAPK: Mitogen-activated protein kinase
PI3K: Phosphoinositide 3-kinase
IC50: The half maximal inhibitory concentration
TUNEL: Terminal deoxynucleotidyl transferase dUTP nick end labeling
SP1: Specificity protein 1
TWIST1: Twist-related protein 1
CHUK: Conserved helix-loop-helix ubiquitous kinase
RELA: Nuclear factor NF-kappa-B p65 subunit
PLA2G4A: Cytosolic phospholipase A2
PTGS2: Prostaglandin-endoperoxide synthase 2
PDE4B: cAMP-specific 3′,5′-cyclic phosphodiesterase 4B
Lum: Luminal
RFS: Recurrence-free survival
ERK: Extracellular signal–regulated kinase
MEK: Mitogen-activated protein kinase kinase
JNK: c-Jun N-terminal kinase
TCGA: The cancer genome atlas
BRCA: Breast cancer
GTEx: Genotype-tissue expression
