# Supplementary material for: Kinase shRNA screening reveals that TAOK3 enhances microtubule-targeted drug resistance of breast cancer cells via the NF-κB signaling pathway
Source: Cell Commun Signal. 2020 Oct 21;18:164. doi: 10.1186/s12964-020-00600-2 (PMC7579951; doi:10.1186/s12964-020-00600-2)

**Supplementary materials**

**Supplementary table captions**

**Supplement Table 1.** IC50 of paclitaxel among breast cancer cell lines.

| **cell lines** | **IC50 (nM)** |
| --- | --- |
| Au565 | 1026.94 |
| T47D | 998.85 |
| BT20 | 35.9508 |
| MDAMB231 | 8.93829 |
| MCF7 | 7.97584 |
| MDAMB453 | 5.35367 |
| MB157 | 2.97242 |
| Hcc38 | 1.69585 |
| BT483 | 1.3269 |
| Hcc70 | 0.79325 |
| Hcc1806 | 0.77282 |
| Hs578t | 0.70671 |
| MDAMB468 | 0.17567 |
| Hcc1143 | 0.15838 |
| Hcc1937 | 0.06745 |

**Supplement Table 2.** Top 50 candidate list from kenome shRNA screening.

| **Symbol** | **Acession no.** | **drug/control** | **p-values** |
| --- | --- | --- | --- |
| FER1L3 | NM_133337 | 0.52016377 | 0.025734 |
| CSNK1A1 | NM_001892 | 0.52314829 | 0.022727 |
| CDK2AP1 | NM_004642 | 0.54413425 | 0.009218 |
| GSK3A | NM_019884 | 0.55099775 | 0.011288 |
| BMX | NM_001721 | 0.57356048 | 0.0346 |
| METTL8 | NM_024770 | 0.58092268 | 0.009238 |
| ABL2 | NM_007314 | 0.58504351 | 0.008138 |
| MARK1 | NM_018650 | 0.58661953 | 0.005986 |
| PDGFRA | NM_006206 | 0.59714639 | 0.02807 |
| PDK2 | NM_002611 | 0.61212094 | 0.000908 |
| SGK3 | NM_019607 | 0.62241756 | 0.006246 |
| METTL6 | NM_152396 | 0.62568057 | 0.000737 |
| TAL2 | NM_005421 | 0.63263604 | 0.00302 |
| METT5D1 | NM_152636 | 0.64245992 | 0.017395 |
| IGF1R | NM_000875 | 0.6430279 | 0.000485 |
| SRC | NM_003930 | 0.64387295 | 0.040775 |
| PRKCB1 | NM_002738 | 0.6675164 | 0.001245 |
| KDR | NM_002253 | 0.66828352 | 0.001245 |
| AKT1 | NM_000476 | 0.67399787 | 0.013704 |
| MARK2 | NM_004954 | 0.6742411 | 0.006196 |
| FGFR1OP | NM_007045 | 0.67745208 | 0.035185 |
| PKR | NM_001135651 | 0.67783229 | 0.002241 |
| TAOK3 | NM_016281 | 0.67999676 | 0.045861 |
| METAP1 | NM_015143 | 0.68240293 | 0.016665 |
| MINK1 | NM_153827 | 0.69121917 | 0.019928 |
| AKT3 | NM_181690 | 0.69413919 | 0.044435 |
| TSSK2 | NM_053006 | 0.70280367 | 0.013033 |
| LCK | NM_005356 | 0.7060116 | 0.008436 |
| MAPK3 | NM_002746 | 0.70633767 | 0.038956 |
| IRAK4 | NM_016123 | 0.70932863 | 0.021514 |
| WNK3 | NM_020922 | 0.70975301 | 0.025703 |
| VRK3 | NM_016440 | 0.71022402 | 0.028046 |
| FGFR1OP2 | NM_015633 | 0.71175264 | 0.013965 |
| FLT3LG | NM_001459 | 0.71679704 | 0.019707 |
| YES1 | NM_005433 | 0.72026176 | 0.00071 |
| AXL | NM_021913 | 0.72189418 | 0.00117 |
| ALK | NM_004304 | 0.72548172 | 0.006706 |
| ABLIM1 | NM_002313 | 0.72594332 | 0.006947 |
| CCL4 | NM_002984 | 0.72960461 | 0.007853 |
| CAMK2N1 | NM_003656 | 0.72967372 | 0.02443 |
| FGFR3 | NM_000142 | 0.73395387 | 0.015389 |
| YSK4 | NM_025052 | 0.73651745 | 0.0058 |
| FLT3 | NM_004119 | 0.73761906 | 0.038268 |
| PRKAR2A | NM_004157 | 0.73929358 | 0.030647 |
| PRKAR2B | NM_002736 | 0.73992413 | 0.00083 |
| TSSK1 | NM_032028 | 0.74101565 | 0.022237 |
| PTK2B | NM_173176 | 0.74318335 | 0.025037 |
| METTL2B | NM_018396 | 0.74405278 | 0.004262 |
| EGFR | NM_005228 | 0.74484424 | 0.001833 |

**Table S3. The fold change of TAOK family in microarray with TAOK3 modulation**

| **probe_ID** | **Symbol** | **FC of TAOK3 OE** | **FC of shTAOK3** |
| --- | --- | --- | --- |
| 216310_at | TAOK1 | 1.2222649 | -1.0407069 |
| 224769_at | TAOK1 | -1.1078191 | 1.052287 |
| 224778_s_at | TAOK1 | 1.0073831 | -1.0384762 |
| 227454_at | TAOK1 | -1.1092539 | -1.8055996 |
| 231193_s_at | TAOK1 | -1.0063448 | 1.1169378 |
| 238420_at | TAOK1 | -1.0088489 | -1.1177961 |
| 204877_s_at | TAOK2 | -1 | 1.161325 |
| 204878_s_at | TAOK2 | 1.0736965 | 1.083649 |
| 204986_s_at | TAOK2 | 1.414719 | 1.2000875 |
| 220761_s_at | TAOK3 | 9.948131 | -6.784412 |
| 221508_at | TAOK3 | -1 | -2.143571 |

**Supplement Figure 1**. **Protein expression TAOK3 and cell viability of paclitaxel in SKBR3** A) The effects of TAOK3 shRNA in Au565, Hcc1806 and SKBR3 and overexpressed TAOK3 in MB157 and Hs578t. B) Paclitaxel sensitivity changes of shTAOK3 SKBR3 cells with paclitaxel treatment.


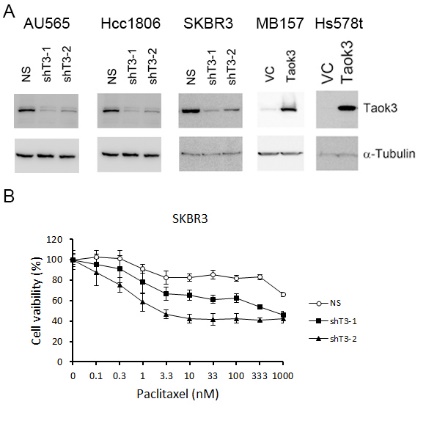


**Supplement Figure 2. The effect of cisplatin and doxorubicin with alternative TAOK3 expression.** A) Cell viability assay of cisplatin among Hcc1806-NS, Hcc1806-shTAOK3-1 and Hcc1806-shTAOK3-2. B) Cell viability assay of cisplatin in Hs578t-VC and Hs578t-TAOK3. C) Cell viability assay of doxorubicin among Hcc1806-NS, Hcc1806-shTAOK3-1 and Hcc1806-shTAOK3-2. B) Cell viability assay of doxorubicin in Hs578t-VC and Hs578t-TAOK3.

**
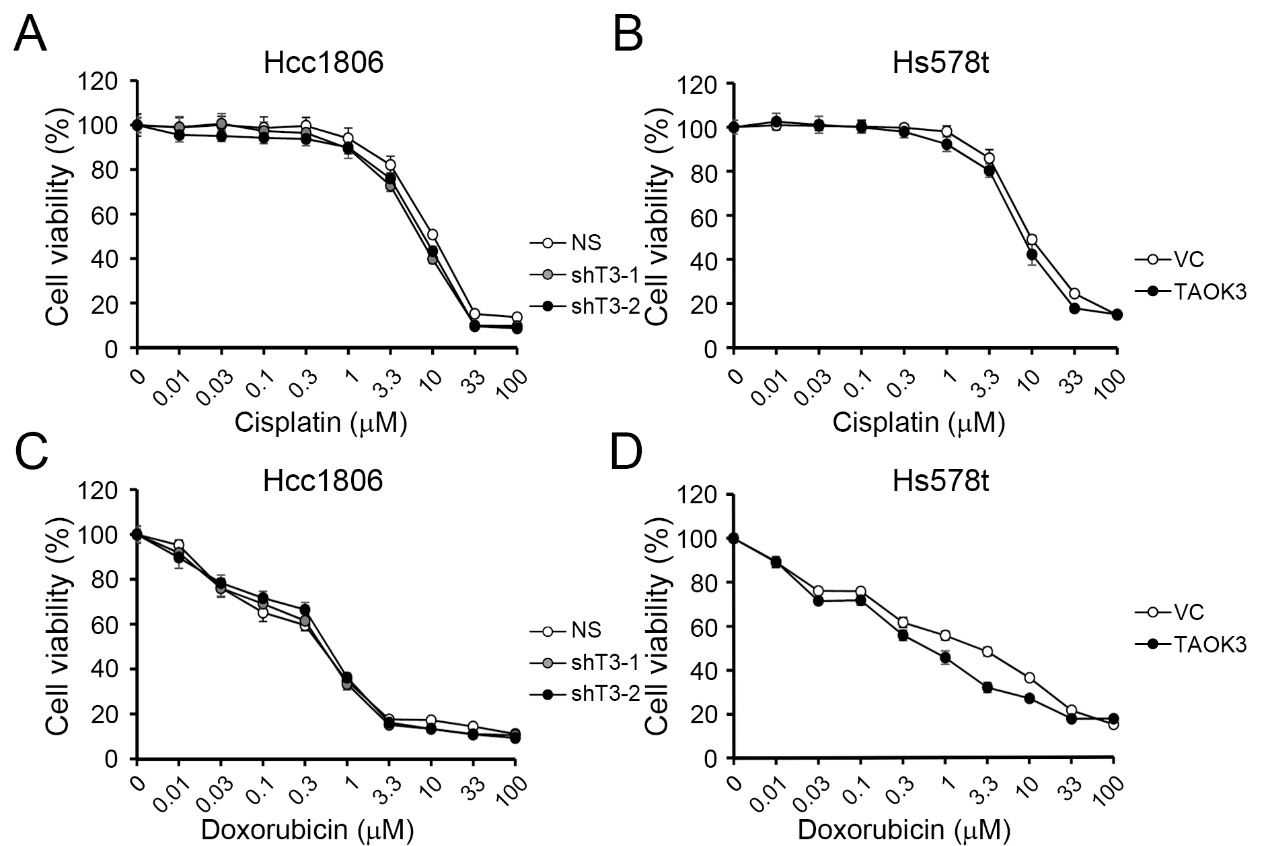
**

**Supplement Figure 3. Growth effects of TAOK3 alternation.** A) Growth curve of shTAOK3 MB157 cells B) Growth curve of TAOK3 overexpression MB156 cells.


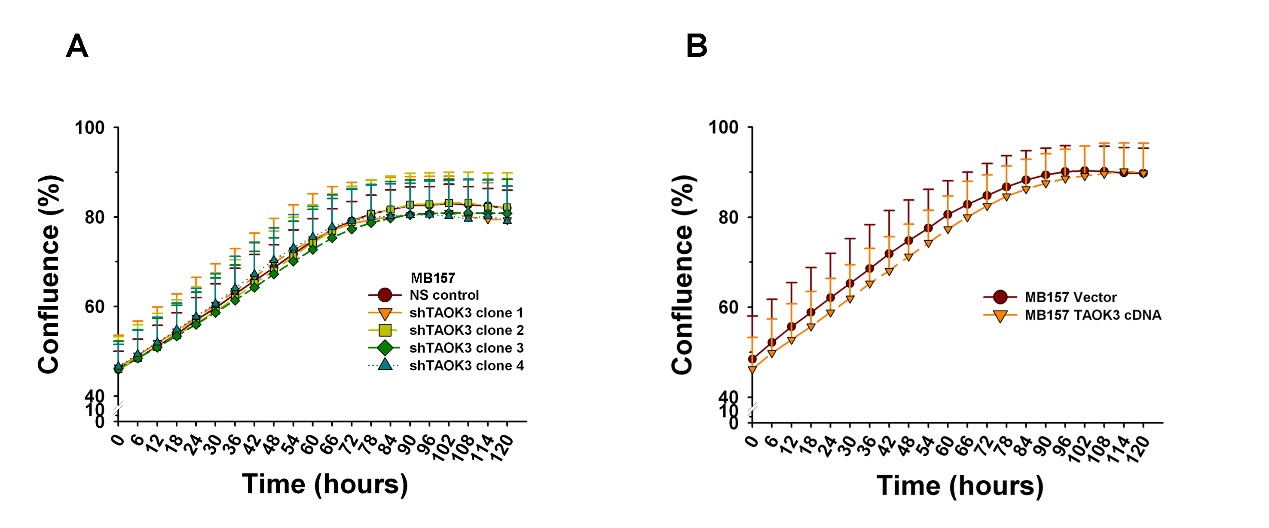


**Supplement Figure 4**. **TUNEL staining in a similar size subcutaneous xenograft tumor of Hs578t-VC, Hs578t-TAOK3, Hcc1806-NS and Hcc1806-shTAOK3 with paclitaxel (Hs578t: 6 mg/kg and Hcc1806: 3 mg/kg) treatment for 24hr.**


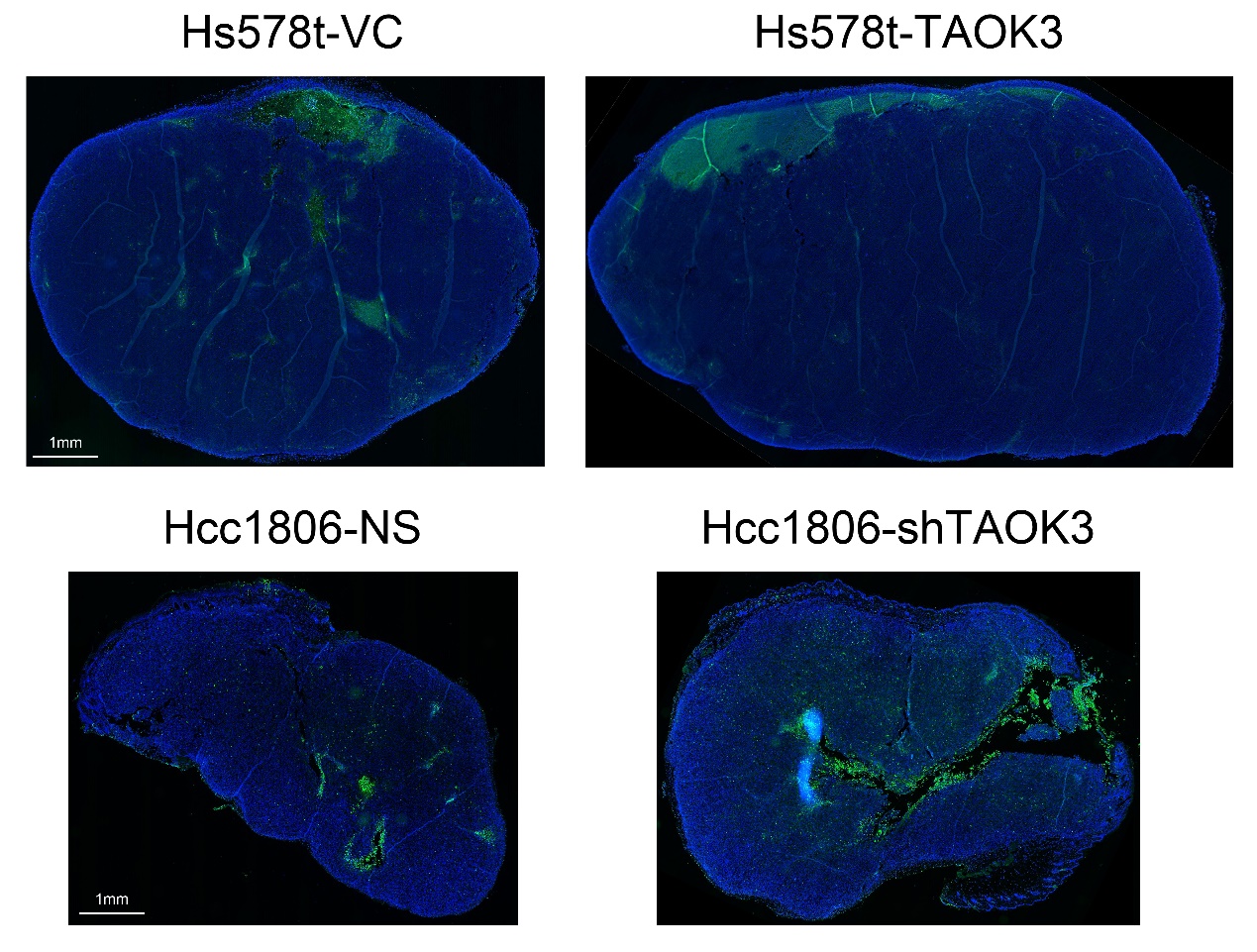


**Supplement Figure 5. Phosphokinase array and microarray analysis of TAOK3 affection.** (A) The bot blot image of phosphoprotein array between Hs578t-VC and Hs578t-TAOK3. (B) Bar chart of top 10 increasing phosphorylated proteins. The semi-quantitation was measured with ImageJ.The network of intersection genes based on upstream analysis in (C) TAOK3 overexpression and (D) shRNA knockdown cells. The number showed the fold change of probe from microarray data.


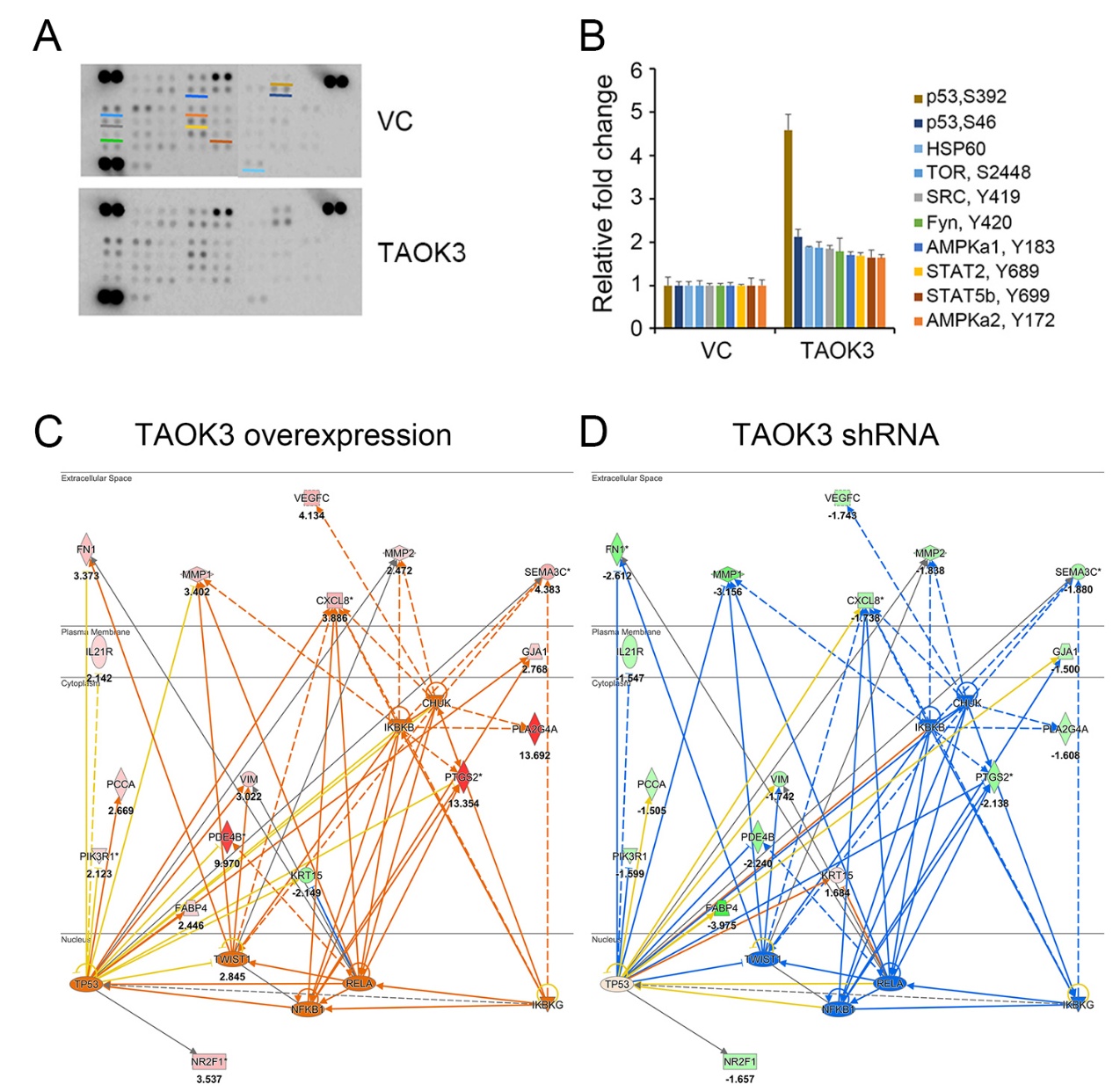


**Supplement Figure 6. The effects of NF-κB shRNAs in Hs578T with TAOK3 modulation cells.** A) The mitotic percentage changes of NF-kB shRNAs and control in Hs578T overexpressed and control cells. B) The cytotoxicity of paclitaxel of NF-κB shRNAs and control in Hs578T control cells.

**
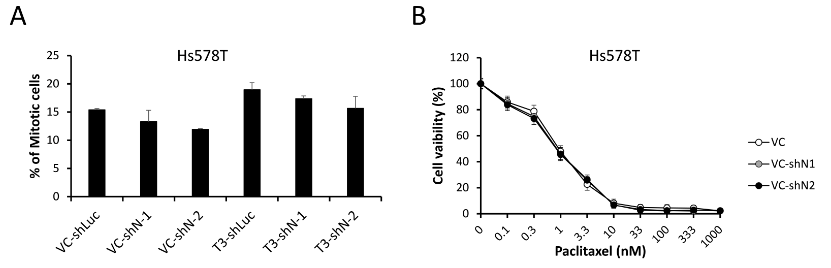
**

**Supplement Figure 7. IHC staining of TAOK3 in xenograft tumor.** Cross-sections of alternative TAOK3 expression xenograft tumor without paclitaxel treatment with TAOK3 IHC staining.


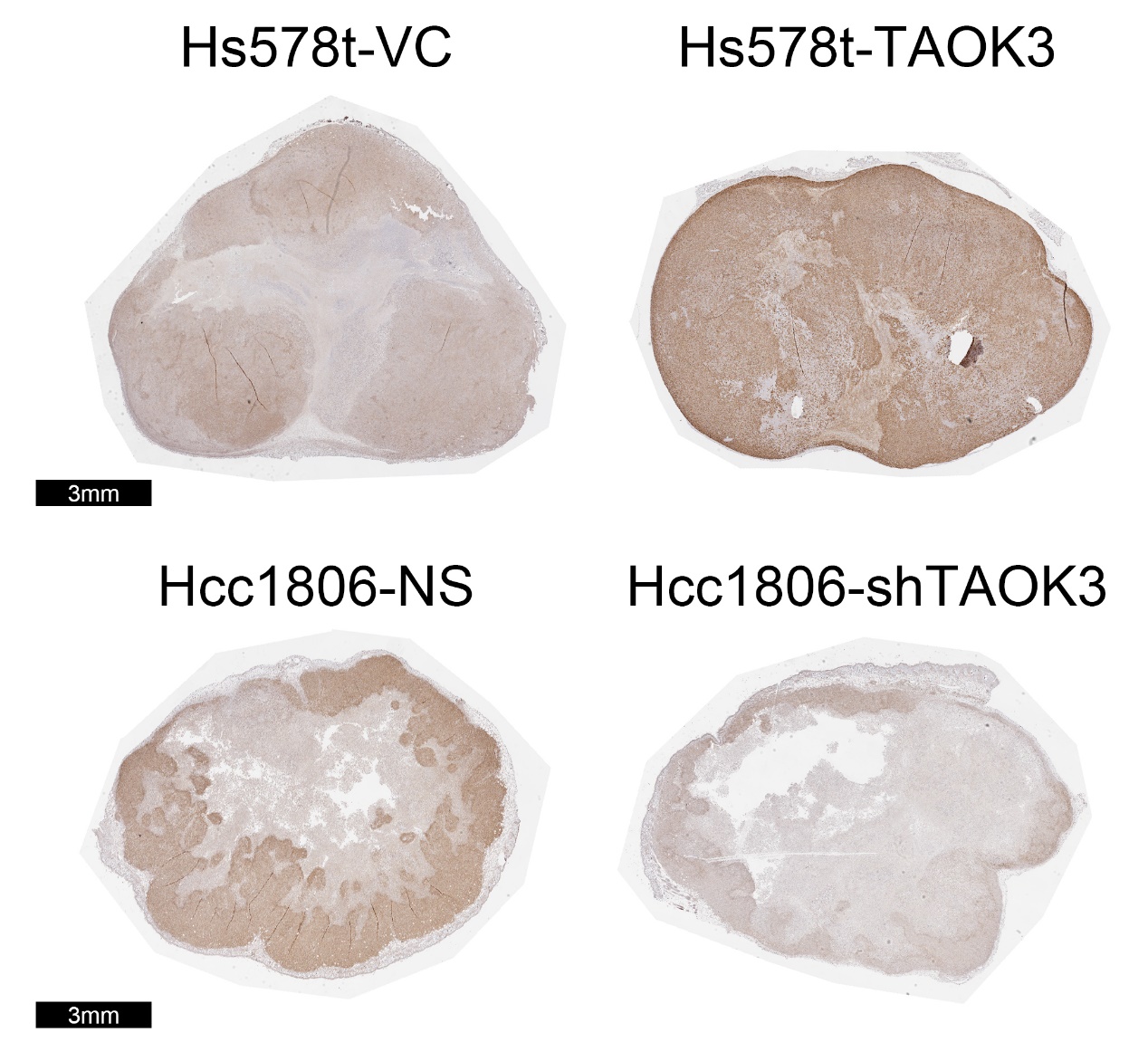

Supplement: Supplementary file 2 — Additional file 1: Supplement Table 1. IC50 of paclitaxel among breast cancer cell lines. Supplement Table 2. Top 50 candidate list from kenome shRNA screening. Supplement Table 3. The fold change of TAOK family in microarray with TAOK3 modulation. Supplement Figure 1. Protein expression TAOK3 and cell viability of paclitaxel in SKBR3 (A) The effects of TAOK3 shRNA in Au565, Hcc1806 and SKBR3 and overexpressed TAOK3 in MB157 and Hs578t. (B) Paclitaxel sensitivity changes of shTAOK3 SKBR3 cells with paclitaxel treatment. Supplement Figure 2. The effect of cisplatin and doxorubicin with alternative TAOK3 expression. (A) Cell viability assay of cisplatin among Hcc1806-NS, Hcc1806-shTAOK3–1 and Hcc1806-shTAOK3–2. (B) Cell viability assay of cisplatin in Hs578t-VC and Hs578t-TAOK3. (C) Cell viability assay of doxorubicin among Hcc1806-NS, Hcc1806-shTAOK3–1 and Hcc1806-shTAOK3–2. (D) Cell viability assay of doxorubicin in Hs578t-VC and Hs578t-TAOK3. Supplement Figure 3. Growth effects of TAOK3 alternation. (A) Growth curve of shTAOK3 MB157 cells (B) Growth curve of TAOK3 overexpression MB157 cells. Supplement Figure 4. TUNEL staining in a similar size subcutaneous xenograft tumor of Hs578t-VC, Hs578t-TAOK3, Hcc1806-NS and Hcc1806-shTAOK3 with paclitaxel (Hs578t: 6 mg/kg and Hcc1806: 3 mg/kg) treatment for 24 h. Supplement Figure 5. Phosphokinase array and microarray analysis of TAOK3 affection. (A) The bot blot image of phosphoprotein array between Hs578t-VC and Hs578t-TAOK3. (B) Bar chart of top 10 increasing phosphorylated proteins. The semi-quantitation was measured with ImageJ.The network of intersection genes based on upstream analysis in (C) TAOK3 overexpression and (D) shRNA knockdown cells. The number showed the fold change of probe from microarray data. Supplement Figure 6. The effects of NF-κB shRNAs in Hs578T with TAOK3 modulation cells. (A) The mitotic percentage changes of NF-B shRNAs and control in Hs578T overexpressed and control cells. (B) The cytotoxicity [file 12964_2020_600_MOESM2_ESM.docx]
